# Supplementary material for: PQBP5/NOL10 maintains and anchors the nucleolus under physiological and osmotic stress conditions
Source: Nat Commun. 2023 Jan 4;14:9. doi: 10.1038/s41467-022-35602-w (PMC9813255; doi:10.1038/s41467-022-35602-w)
Supplement: Supplementary file 1 — Supplementary Information [file 41467_2022_35602_MOESM1_ESM.pdf]

Supplementary Information

PQBP5/NOL10 maintains and anchors the nucleolus under physiological and osmotic stress conditions

Xiaocen Jin[1,#], Hikari Tanaka[1,#], Meihua Jin[1], Kyota Fujita[1], Hidenori Homma[1], Maiko Inotsume[1], Huang Yong[1], Kenichi Umeda[2], Noriyuki Kodera[2], Toshio Ando[2] and Hitoshi Okazawa[1, 3, $]

1: Department of Neuropathology, Medical Research Institute, Tokyo Medical and Dental University, 1-5-45, Yushima, Bunkyo-ku, Tokyo 113-8510, Japan
2: Nano Life Science Institute, Kanazawa University, Kakuma-machi, Kanazawa, Ishikawa 920-1192, Japan
3: Center for Brain Integration Research, Tokyo Medical and Dental University, 1-5-45, Yushima, Bunkyo-ku, Tokyo 113-8510, Japan

#: These authors contributed equally.
$: Correspondence should be addressed to H.O.
E-mail: okazawa-tky@umin.ac.jp

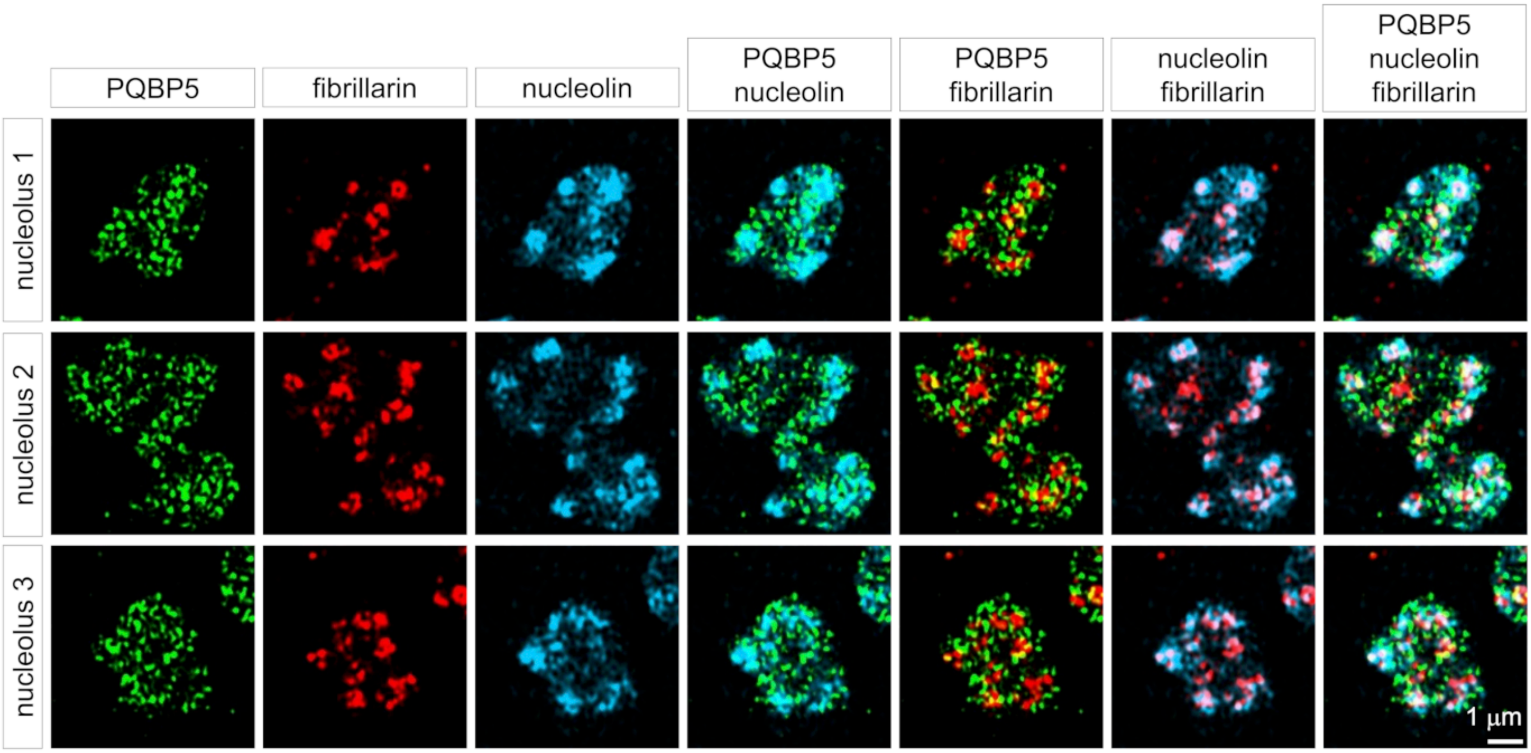

Supplementary Figure 1
Super resolution microscopy analysis of PQBP5 and other proteins in nucleolus
Super resolution microscopy images of three nucleoli. The distribution patterns of PQBP5, fibrillarin, and nucleolin were similar to those shown in Figure 1d-f. The similar observation was repeated more than ten times.

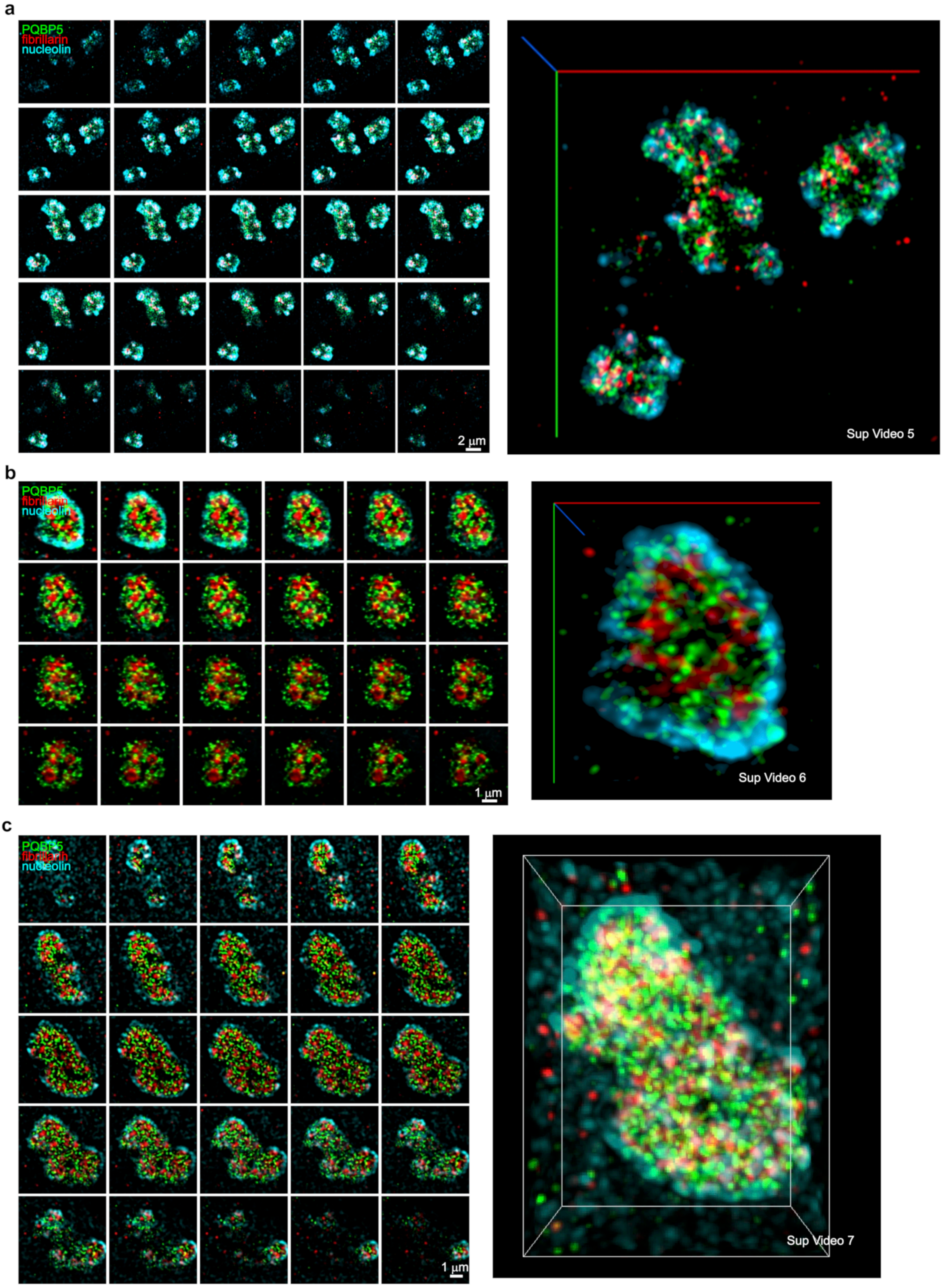

a
PQBP5
fibrillarin
nucleolin
2 µm
Sup Video 5
b
PQBP5
fibrillarin
nucleolin
1 µm
Sup Video 6
c
PQBP5
fibrillarin
nucleolin
1 µm
Sup Video 7

Supplementary Figure 2
Source images for 3D reconstruction
Source images for Supplementary Videos 5–7. Right images are 2D projections of 3D reconstructed images.
a) Images obtained by LSM980 with Airyscan 2 (Carl Zeiss Co., Ltd).
b) Images obtained by Elyra 7 (Carl Zeiss Co., Ltd).
c) Images obtained by CSU-W1 SoRa (Yokogawa Electronic Corporation).
The similar observation was repeated more than five times.

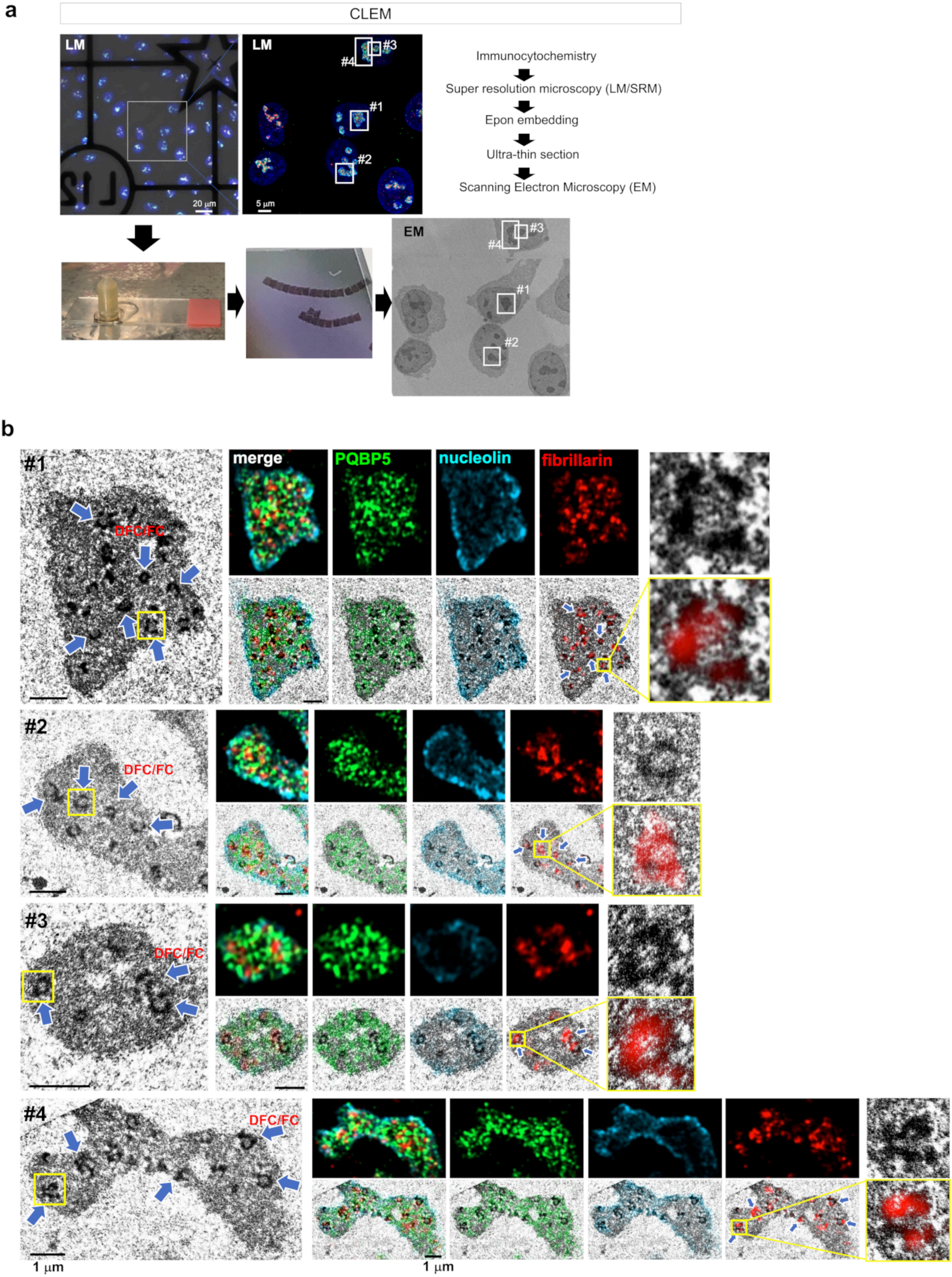
a
CLEM
LM
LM
20 µm
5 µm
#1
#2
#3
#4
Immunocytochemistry
Super resolution microscopy (LM/SRM)
Epon embedding
Ultra-thin section
Scanning Electron Microscopy (EM)
EM
b
merge
PQBP5
nucleolin
fibrillarin
DFC/FC
1 µm
1 µm

Supplementary Figure 3
CLEM analysis of nucleolar proteins
a) Super resolution microscopy images of HeLa cells on the grid analyzed by immunocytochemistry. Serial ultrathin sections were subsequently analyzed by scanning electron microscopy. Detailed information is provided in the Methods.
b) CLEM analysis of four nucleoli confirming fibrillarin localization to the DFC. The similar experiment was repeated three times.

a

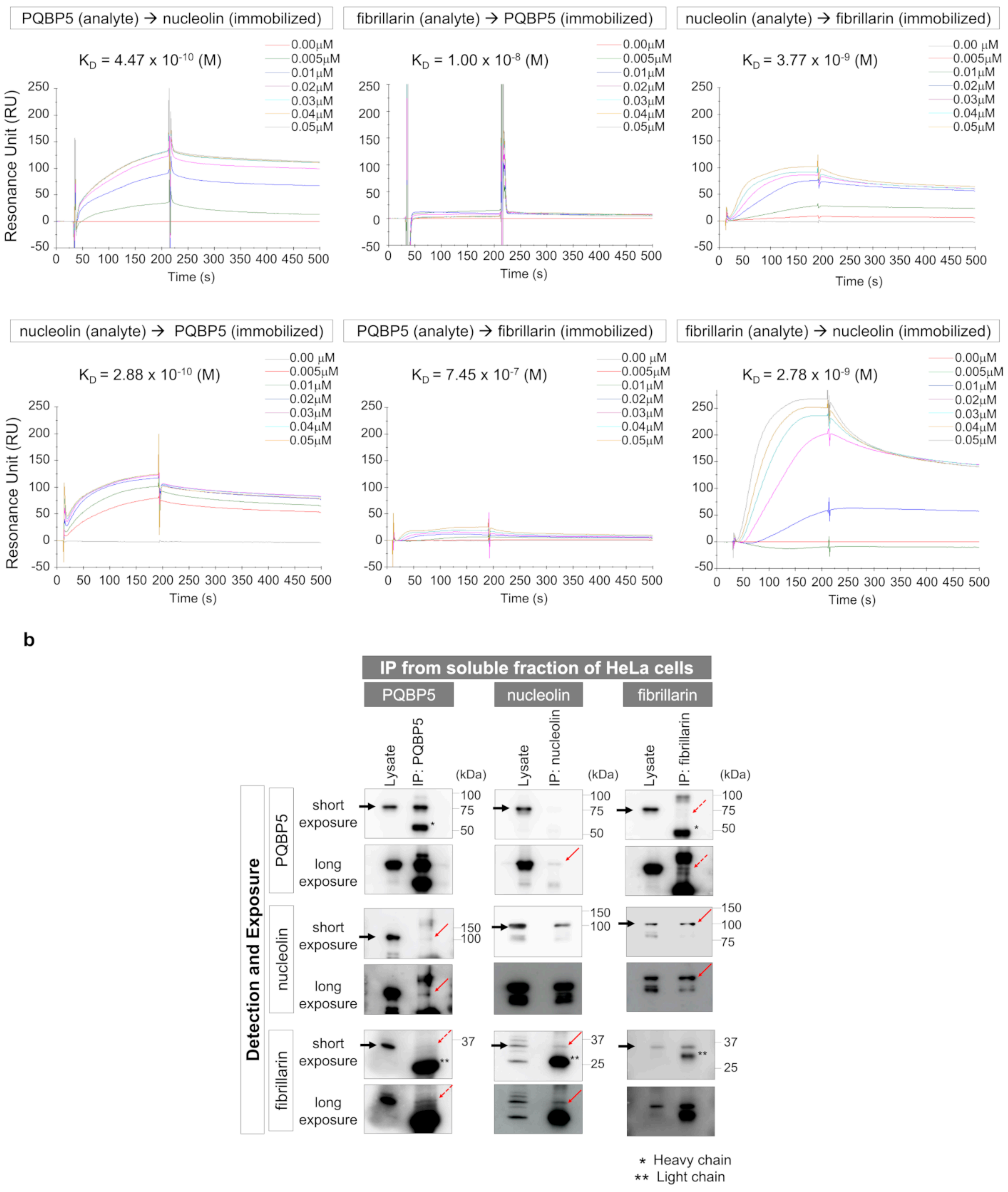

Supplementary Figure 4
Interaction between PQBP5 and other nucleolar proteins
a) Surface plasmon resonance analyses of the pairwise interactions of PQBP5, fibrillarin, and nucleolin.
b) Immunoprecipitation analyses of pairwise interactions of PQBP5, fibrillarin, and nucleolin. Weak interactions were detected between PQBP5 and nucleolin and between nucleolin and fibrillarin (red arrows), but not between PQBP5 and fibrillarin (red dot arrows). The similar experiment was repeated three times.

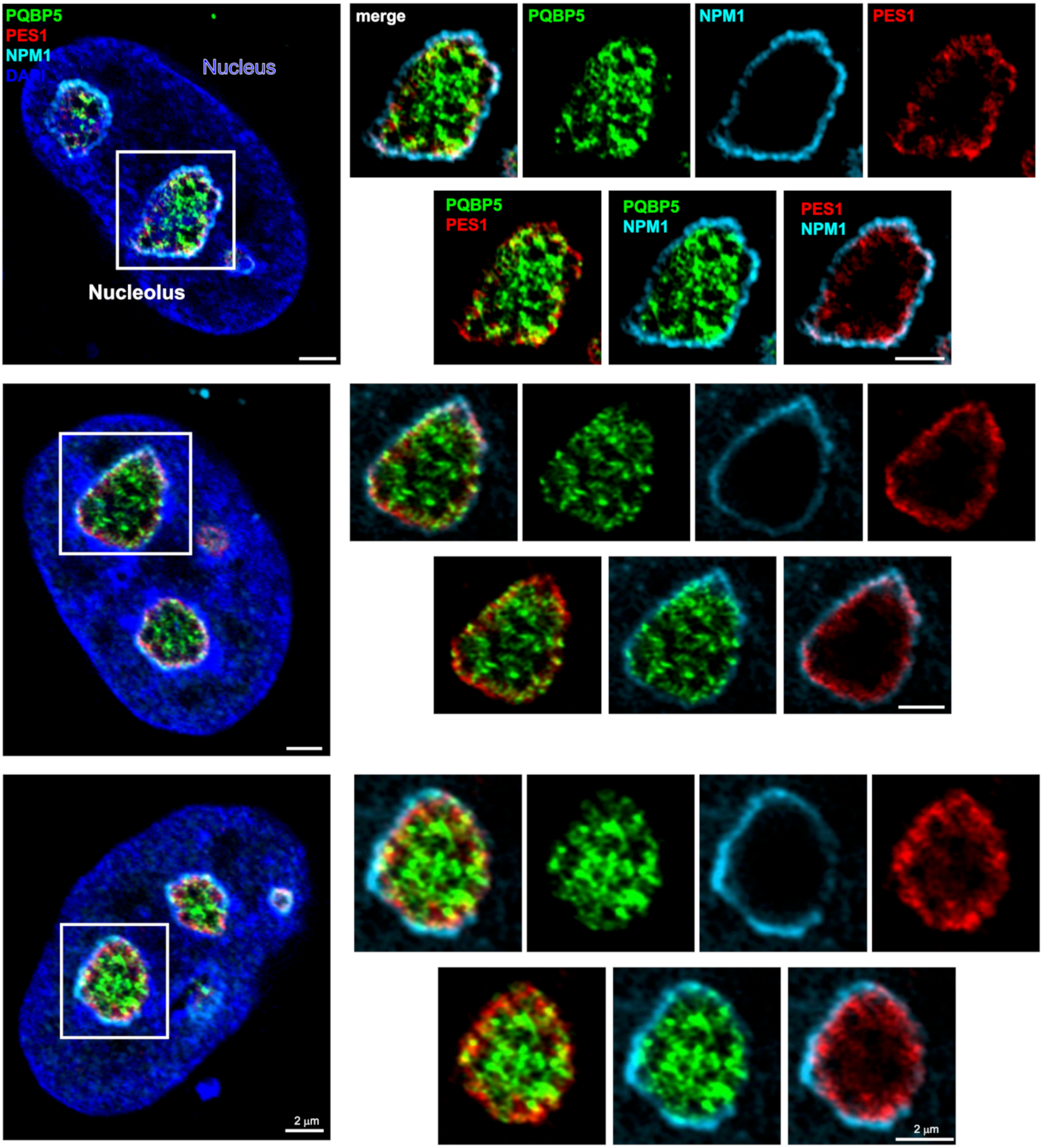

Supplementary Figure 5
Super resolution microscopy analysis of PQBP5, NPM1 and PES1 in the nucleolus
Super resolution microscopic analyses of HeLa cells showing that NPM1 and PES1 were distributed at the periphery of the nucleolus, forming lamellar outer shells, whereas PQBP5 formed a meshwork structure constituting the core of the nucleolus. The similar experiment was repeated three times.

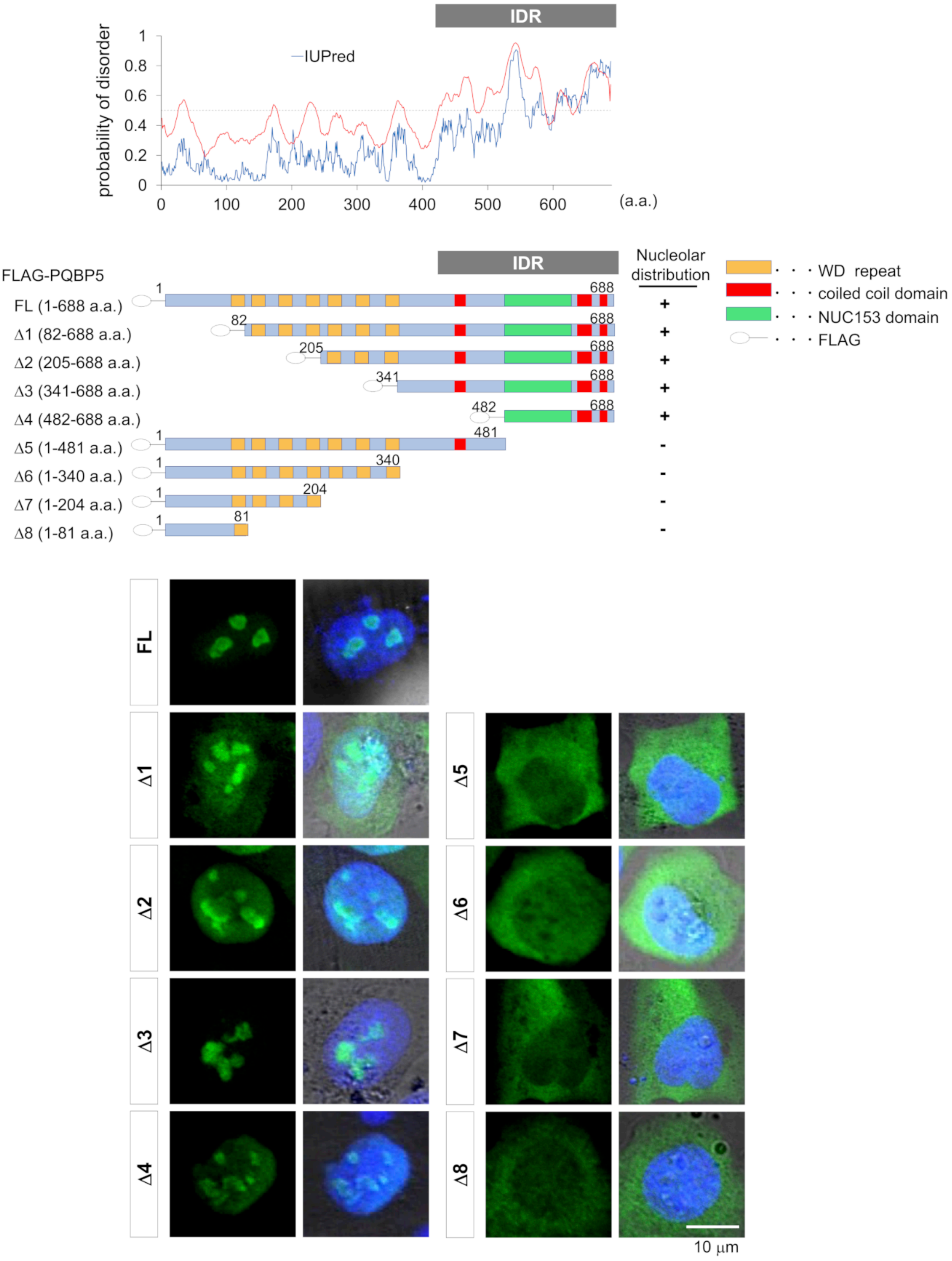

Supplementary Figure 6

IDR is essential for nucleolar localization of PQBP5

Deletion of FLAG-PQBP5 fusion protein to determine the essential domain(s) for the nucleolar localization of PQBP5. Immunocytochemistry with anti-FLAG antibody, performed 2 days after transfection of the plasmid into U2OS cells, revealed that IDR, especially the NUC153 domain, was essential for nucleolar localization. The similar experiment was repeated three times.

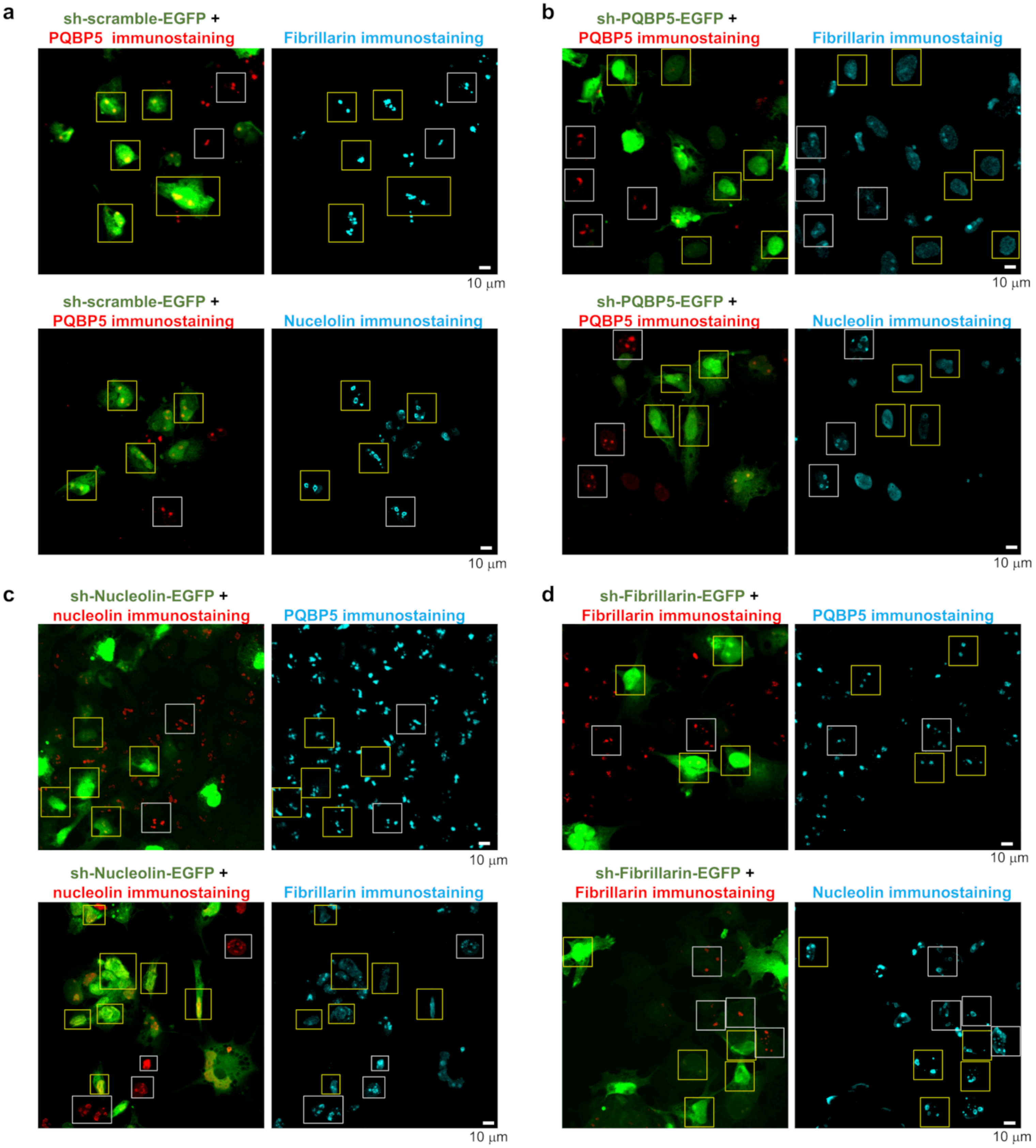

Supplementary Figure 7
Source images for the analysis in Figure 2
Low-magnification images obtained by knockdown experiments investigating the hierarchy of PQBP5, nucleolin, and fibrillarin.
a) HeLa cells transfected with sh-scramble-EGFP immunostained with antibodies to PQBP5 and fibrillarin or nucleolin.
b) HeLa cells transfected with sh-PQBP5-EGFP immunostained with antibodies to PQBP5 and fibrillarin or nucleolin.
c) HeLa cells transfected with sh-nucleolin-EGFP immunostained with antibodies to nucleolin and PQBP5 or fibrillarin.
d) HeLa cells transfected with sh-fibrillarin-EGFP immunostained with antibodies to fibrillarin and PQBP5 or nucleolin.
The similar experiment was repeated four times.

Supplementary Figure 8

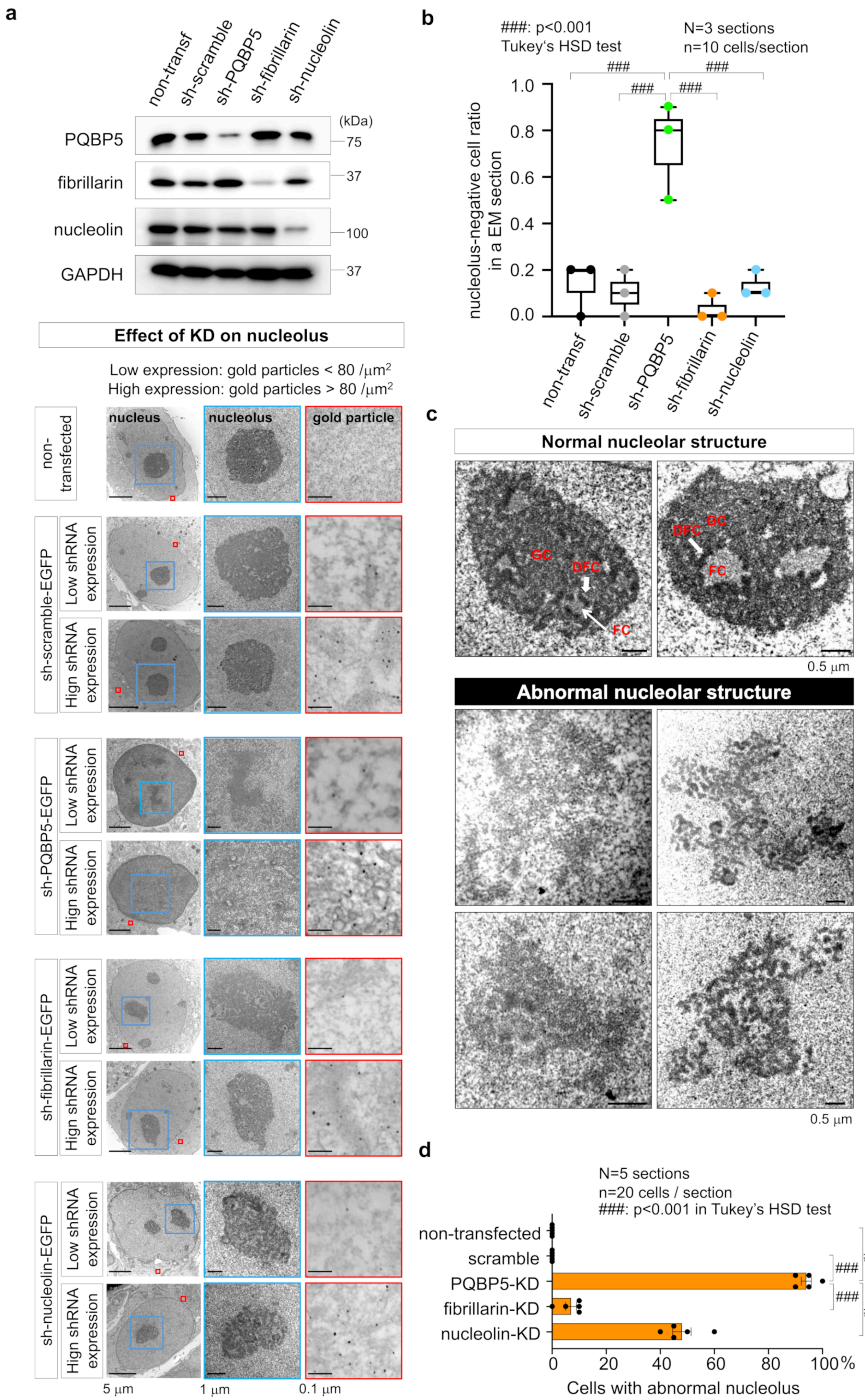

Supplementary Figure 8

Electron microscopic analysis of the effects of nucleolar protein knockdown on nucleolus structure

a) Effect of shRNA-mediated knockdown of a nucelolar protein on the nucleolus, as determined by immunoelectron microscopy with an anti-EGFP antibody. Confirmation of the presence of gold particles attached to the secondary antibody (right panels) in the region indicated by the red square (left panels) of HeLa cells expressing shRNA. Middle panels show enlargement of a nucleolus in a blue square (left panels). Cells were classified as low and high expressers of shRNA based on the density of gold particles. PQBP5 knockdown changed the structure of the nucleolus in an shRNA-dose-dependent manner, whereas knockdown of fibrillarin or nucleolin did not, although minor changes in nucleolus structure, such as an irregular shape, were observed. The similar experiment was repeated three times.

b) Percentages of nucleolus-negative cells among all cells in three EM sections. Box plot shows median, 25-75th percentile, and whiskers representing data outside the 25-75th percentile range. Tukey's HSD test was used for multiple comparisons. P-values are 0.0009 in non-transfected vs sh-PQBP5, 0.0006 in sh-scramble vs sh-PQBP5, 0.0003 in sh-fibrillarin vs sh-PQBP5, and 0.0009 in sh-nucleolin vs sh-PQBP5.

c) Morphological changes in the nucleolus. Abnormal nucleoli showed features such as irregular shape, low electron density, and no FC/DFC unit. The similar experiment was repeated five times.

d) Percentages of abnormal cells with abnormal changes in nucleolar morphology among gold-particle-positive cells expressing shRNA-EGFP. Data are presented as mean values +/- SEM. Tukey's HSD test was used for multiple comparisons. $P<0.0001$ in comparisons by GraphPad Prism.

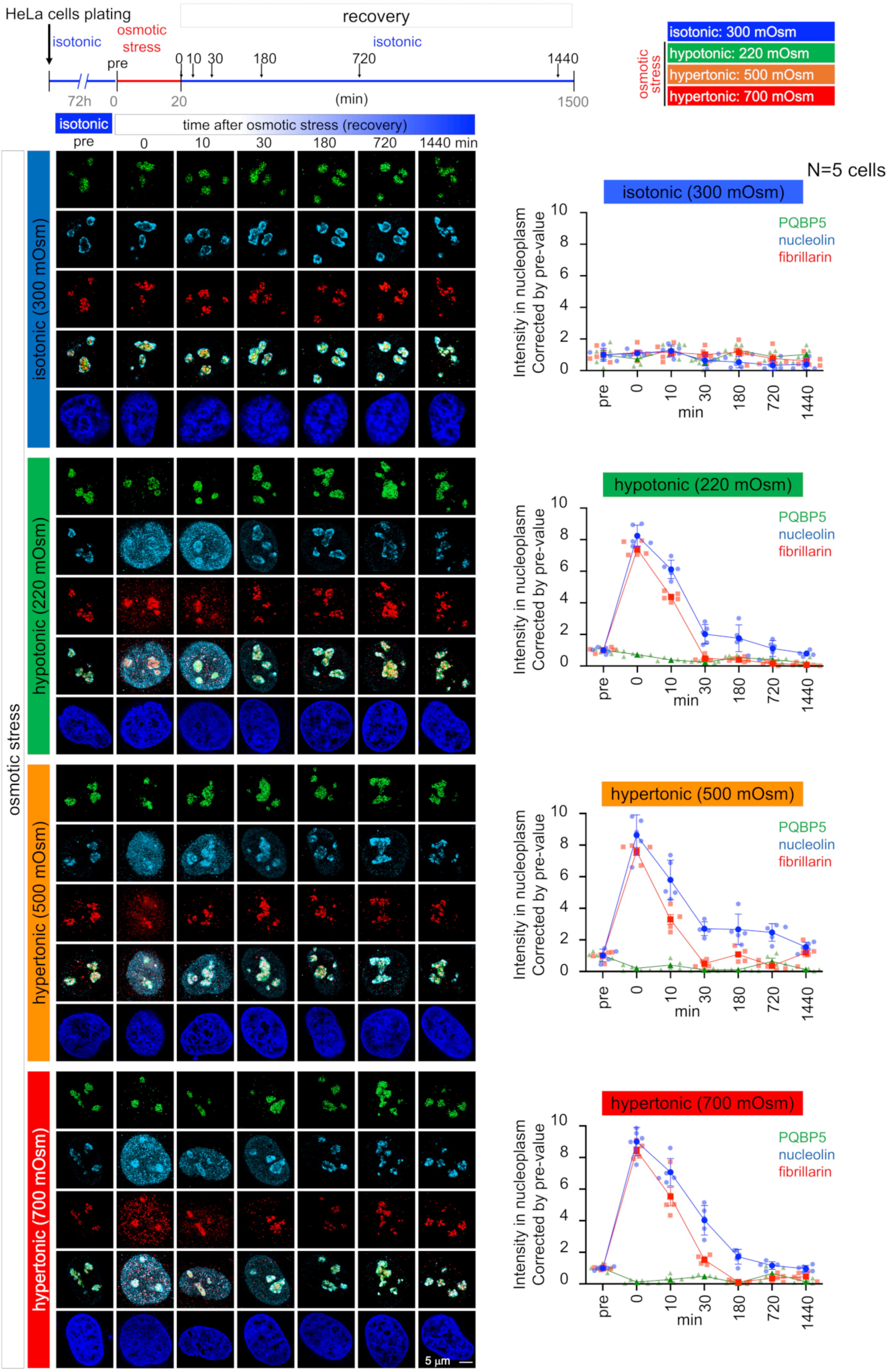
HeLa cells plating
isotonic
osmotic stress
pre
recovery
isotonic
0 10 30 180 720 1440
72h 0 20 (min) 1500
isotonic: 300 mOsm
hypotonic: 220 mOsm
hypertonic: 500 mOsm
hypertonic: 700 mOsm
osmotic stress
isotonic
time after osmotic stress (recovery)
pre 0 10 30 180 720 1440 min
osmotic stress
isotonic (300 mOsm)
hypotonic (220 mOsm)
hypertonic (500 mOsm)
hypertonic (700 mOsm)
5 µm
N=5 cells
Intensity in nucleoplasm Corrected by pre-value
PQBP5
nucleolin
fibrillarin
pre 0 10 30 180 720 1440
min

Supplementary Figure 9

Super resolution microscopic analysis of nucleolar proteins under osmotic stress conditions

Super resolution microscopy analysis of the morphological and quantitative changes of the three nucleolar proteins during and after osmotic stress in a single cell. The right graphs show the relative signal intensities of fibrillarin and nucleolin in the nucleoplasm outside the nucleoli during and after osmotic stress. Data are presented as mean values +/- SEM.

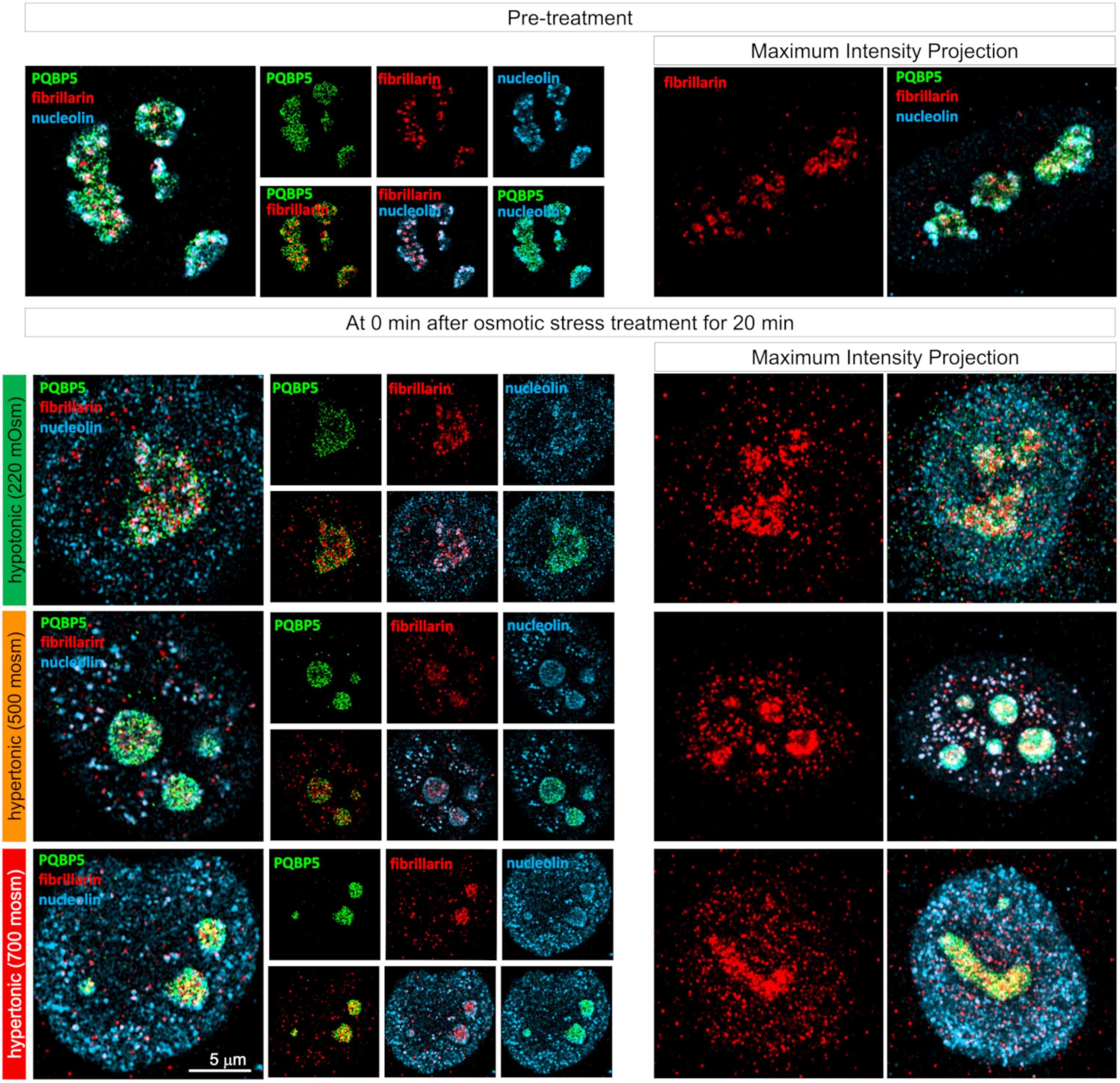

Supplementary Figure 10
Higher magnification of super resolution microscopic images of nucleoli under stress
High magnification images of HeLa cells obtained by super resolution microscopy before (upper panels) and immediately after (lower panels) osmotic stress treatments. Images during the early phase of recovery showed rapid assembly of fibrillarin to PQBP5 foci, with some nucleolin remaining in the nucleoplasm outside the nucleolus. The upper right and lower right panels show images obtained by the maximum intensity projection methods, more clearly showing the dispersion of fibrillarin in response to osmotic stress. The similar experiment was repeated four times.

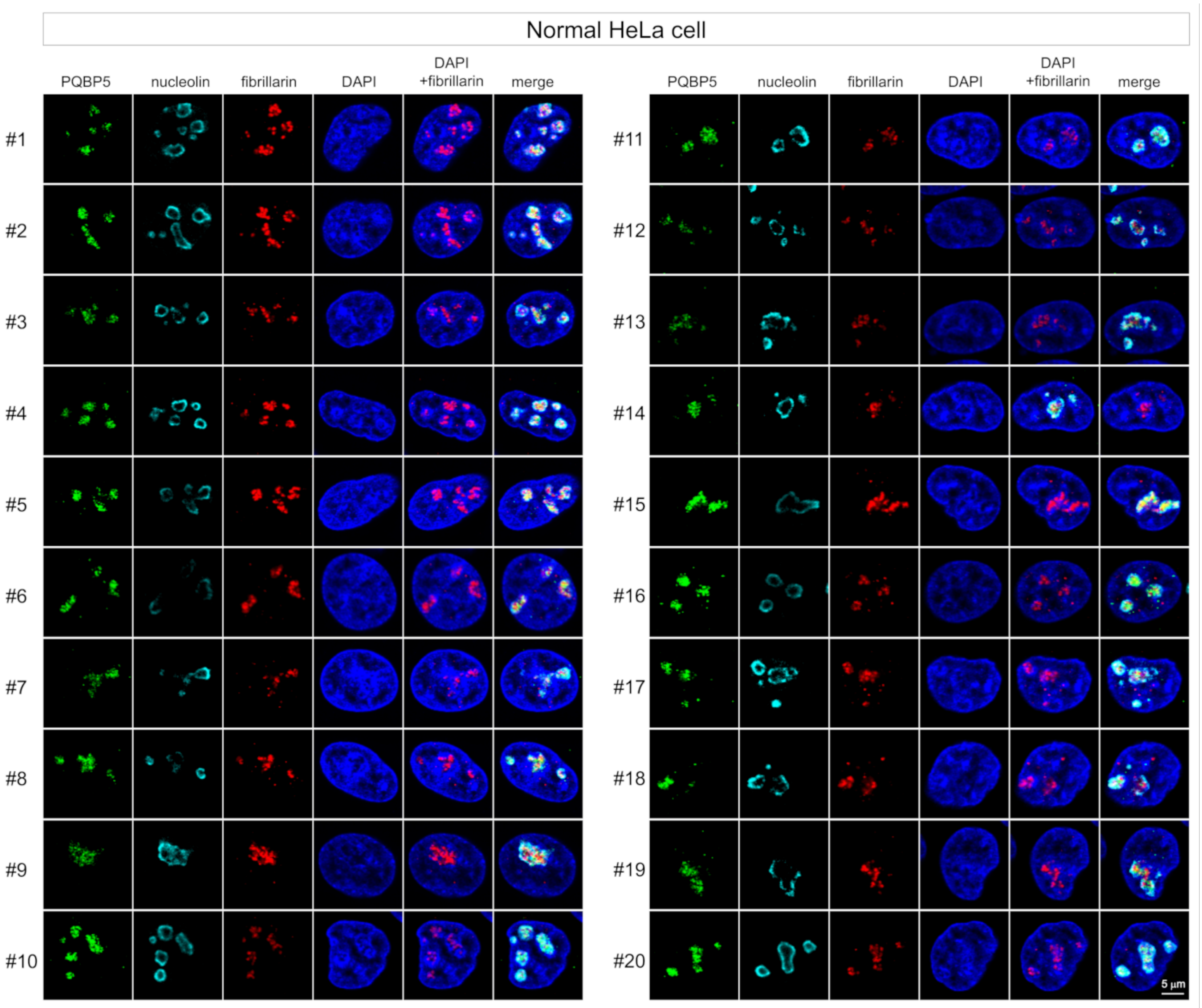

Supplementary Figure 11
Relationship between DAPI and nucleolar protein staining patterns in normal HeLa cells
Images of 20 normal HeLa cells stained with DAPI and antibodies against nucleolar proteins. Nucleoli tended to be located in DAPI-negative areas surrounded by high DAPI signals, although many exceptions were observed. Therefore, accurate prediction of nucleoli solely by DAPI staining is basically impossible. The similar experiment was repeated three times.

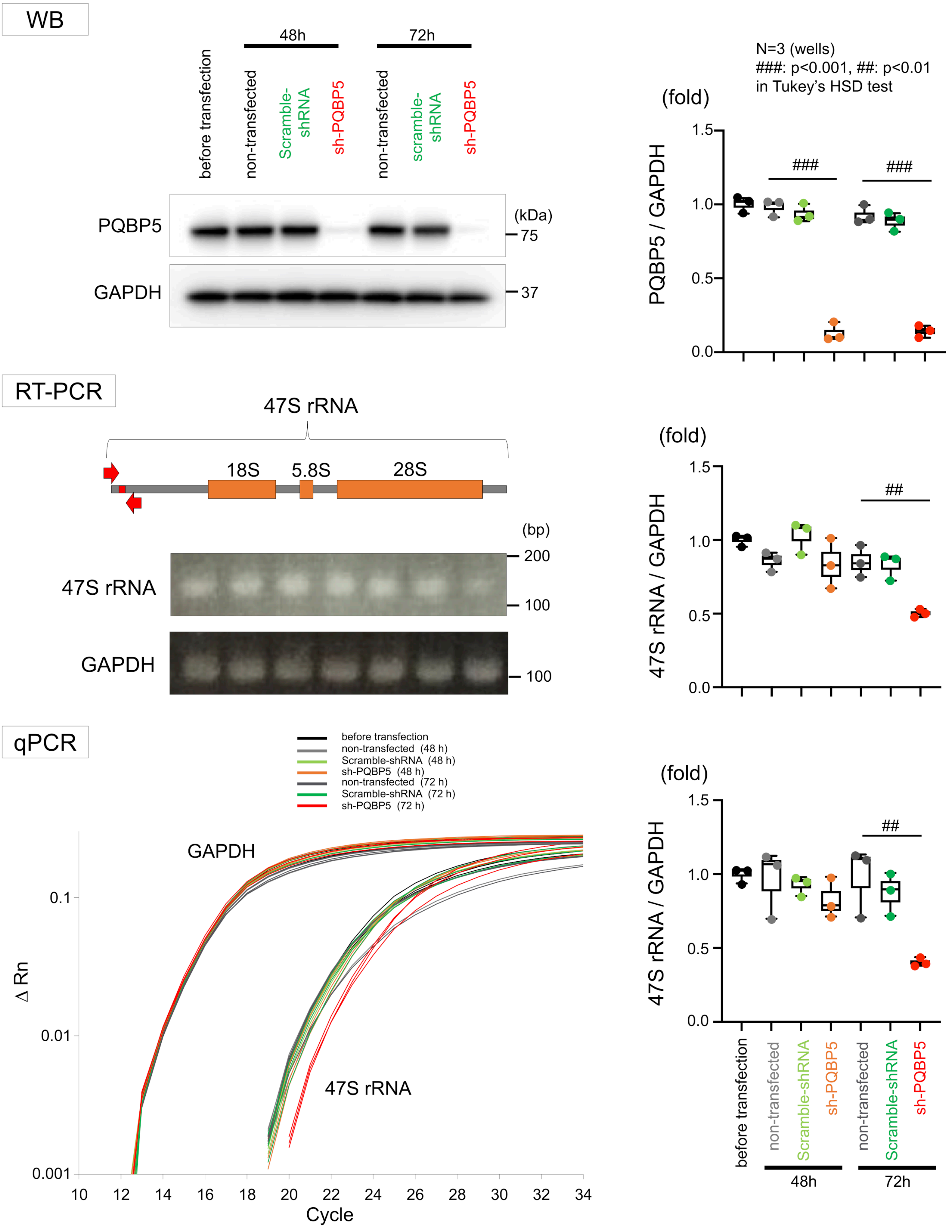
WB
48h
72h
before transfection
non-transfected
Scramble-shRNA
sh-PQBP5
non-transfected
scramble-shRNA
sh-PQBP5
PQBP5
GAPDH
(kDa)
75
37
N=3 (wells)
###: p<0.001, ##: p<0.01
in Tukey's HSD test
(fold)
PQBP5 / GAPDH
###
###
RT-PCR
47S rRNA
18S
5.8S
28S
(bp)
200
100
47S rRNA
GAPDH
100
(fold)
47S rRNA / GAPDH
##
qPCR
before transfection
non-transfected (48 h)
Scramble-shRNA (48 h)
sh-PQBP5 (48 h)
non-transfected (72 h)
Scramble-shRNA (72 h)
sh-PQBP5 (72 h)
GAPDH
47S rRNA
Δ Rn
Cycle
(fold)
47S rRNA / GAPDH
##
before transfection
non-transfected
Scramble-shRNA
sh-PQBP5
non-transfected
Scramble-shRNA
sh-PQBP5
48h
72h

Supplementary Figure 12

Chronological changes of 47S rRNA, PQBP5, and GAPDH levels following PQBP5 knockdown

Western blot and qPCR (semi-quantitative and quantitative RT-PCR) analyses of HeLa cell samples before and 48 and 72 hours after transfection of PQBP5-shRNA plasmid. Collected cells were divided for preparation of protein and RNA samples. qPCR of 47S rRNA and western blot of PQBP5 and GAPDH were performed as described in Methods. 47S rRNA, which is promptly processed to 18S, 5.8S, and 28S rRNA molecules, reflects the real-time Pol I transcription level. Box plot shows median, 25-75th percentile, and whiskers representing data outside the 25-75th percentile range. Tukey's HSD test was used for multiple comparisons. In western blot, *P*-values by GraphPad Prism are less than 0.0001 in comparison of PQBP5/GAPDH in non-transfected vs sh-PQBP5 at 48 hours or 72 hours after transfection. In RT-PCR, *P*-value is 0.0095 in comparison of 47S rRNA/GAPDH in non-transfected vs sh-PQBP5 at 72 hours after transfection. In qPCR, *P*-value is 0.0050 in comparison of 47S rRNA/GAPDH in non-transfected vs sh-PQBP5 at 72 hours after transfection.

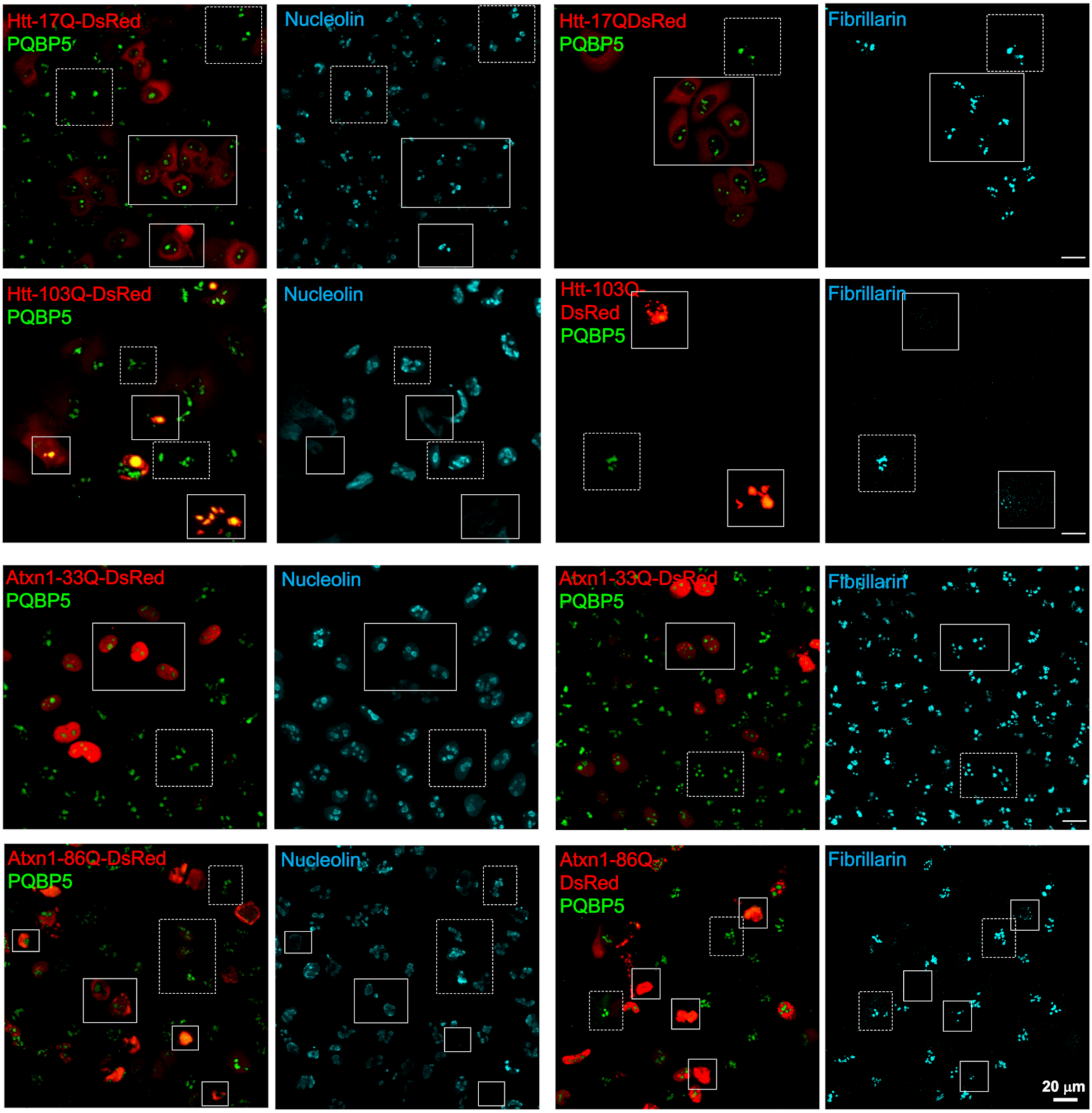

Supplementary Figure 13

Low magnification of U2OS cells transfected with plasmids expressing normal and mutant Htt and Atxn1 proteins

DsRed-positive transfected cells expressing normal Htt-17Q/Atxn1-33Q proteins (white square), showing normal distributions of nucleolin and fibrillarin, similar to DsRed-negative non-transfected cells (dotted white square). By contrast, DsRed-positive transfected cells expressing mutant Htt-103Q/Atxn1-86Q proteins (white square) showed abnormal distribution or lack of expression of nucleolin and fibrillarin, findings distinct from those of DsRed-negative non-transfected cells (dotted white square).

The similar experiment was repeated four times.

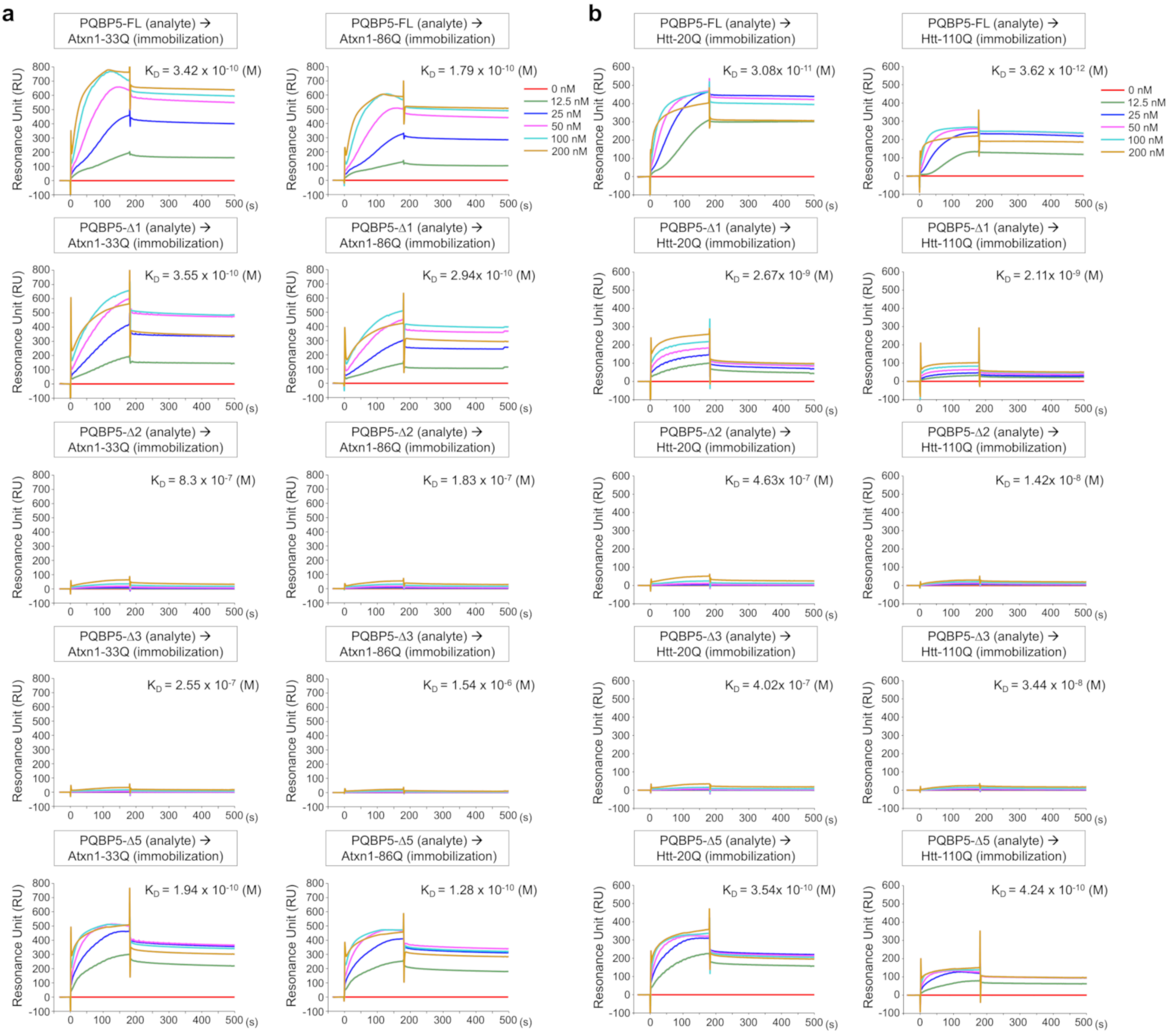

Supplementary Figure 14

SPR analysis of affinities of polyglutamine disease proteins for PQBP5

a) SPR analysis of interactions between normal/mutant Atxn1 and full-length (FL)/deletion mutant PQBP5.

b) SPR analysis of interactions between normal/mutant Htt and full-length (FL)/deletion mutant PQBP5.

The WD domains of PQBP5 were essential for interactions with Atxn1 and Htt. Moreover, both normal and mutant Atxn1/Htt interacted with PQBP5.

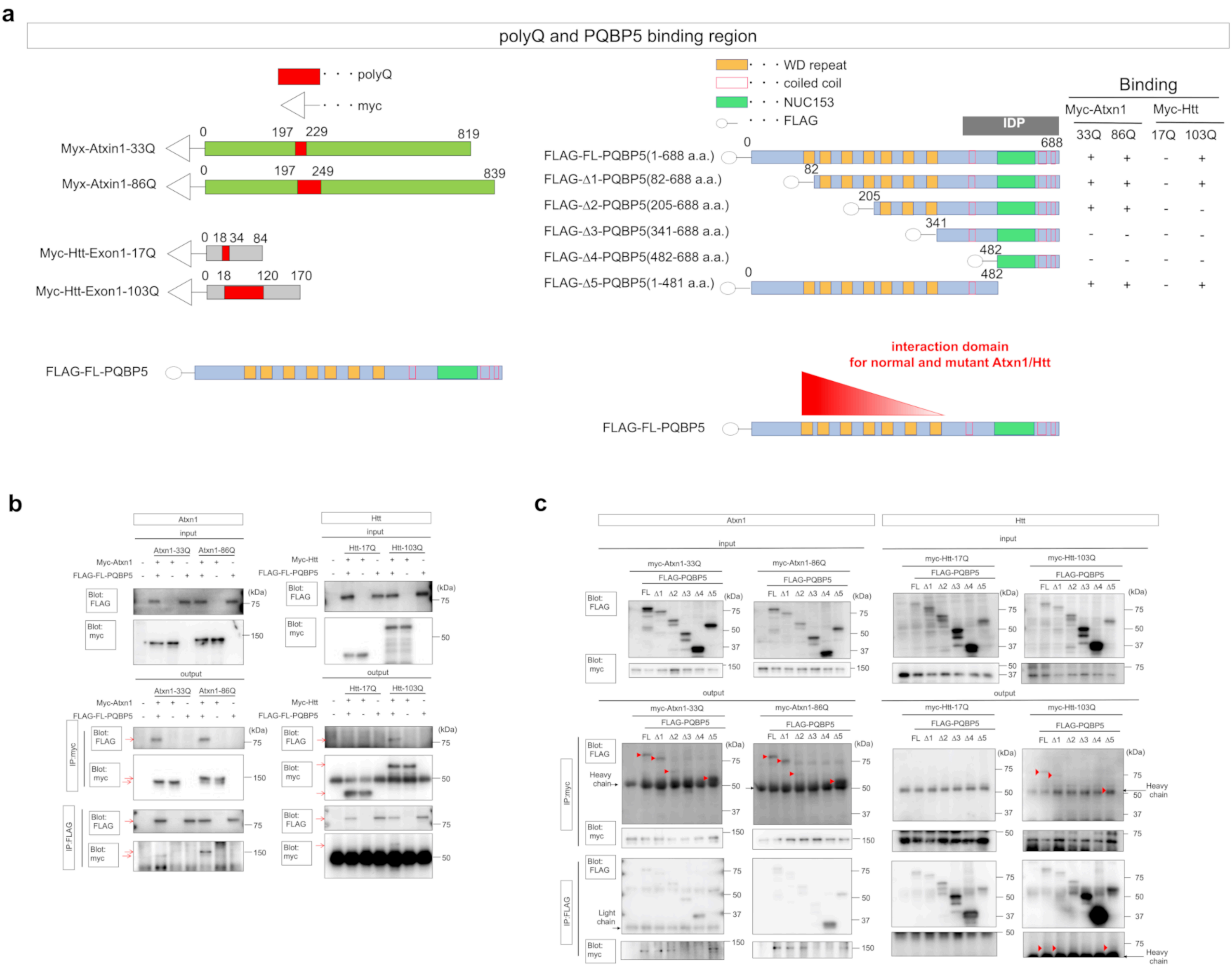

Supplementary Figure 15

Immunoprecipitation analysis of interactions of polyglutamine disease proteins with PQBP5

a) Full-length (FL) and deletion mutants of PQBP5 (upper scheme) were used in immunoprecipitation analysis to determine the domains of PQBP5 that interact with normal/mutant Atxn1 and Htt.

b) Immunoprecipitation analyses of HeLa cells transfected with FLAG-FL-PQBP5 and Myc-Atxn1 or Myc-Htt protein reveals interaction of full-length PQBP5 with normal/mutant Atxn1 or with normal/mutant Htt. HeLa cells expressing only FLAG-PQBP5 or only Myc-polyQ protein. The similar experiment was repeated three times.

c) Immunoprecipitation analyses of HeLa cells transfected with FLAG-FL-PQBP5 and deletion mutants of Myc-Atxn1 or Myc-Htt protein, showing that mutants lacking WD40 repeats lose their affinities for Atxn1 and Htt. The similar experiment was repeated three times.

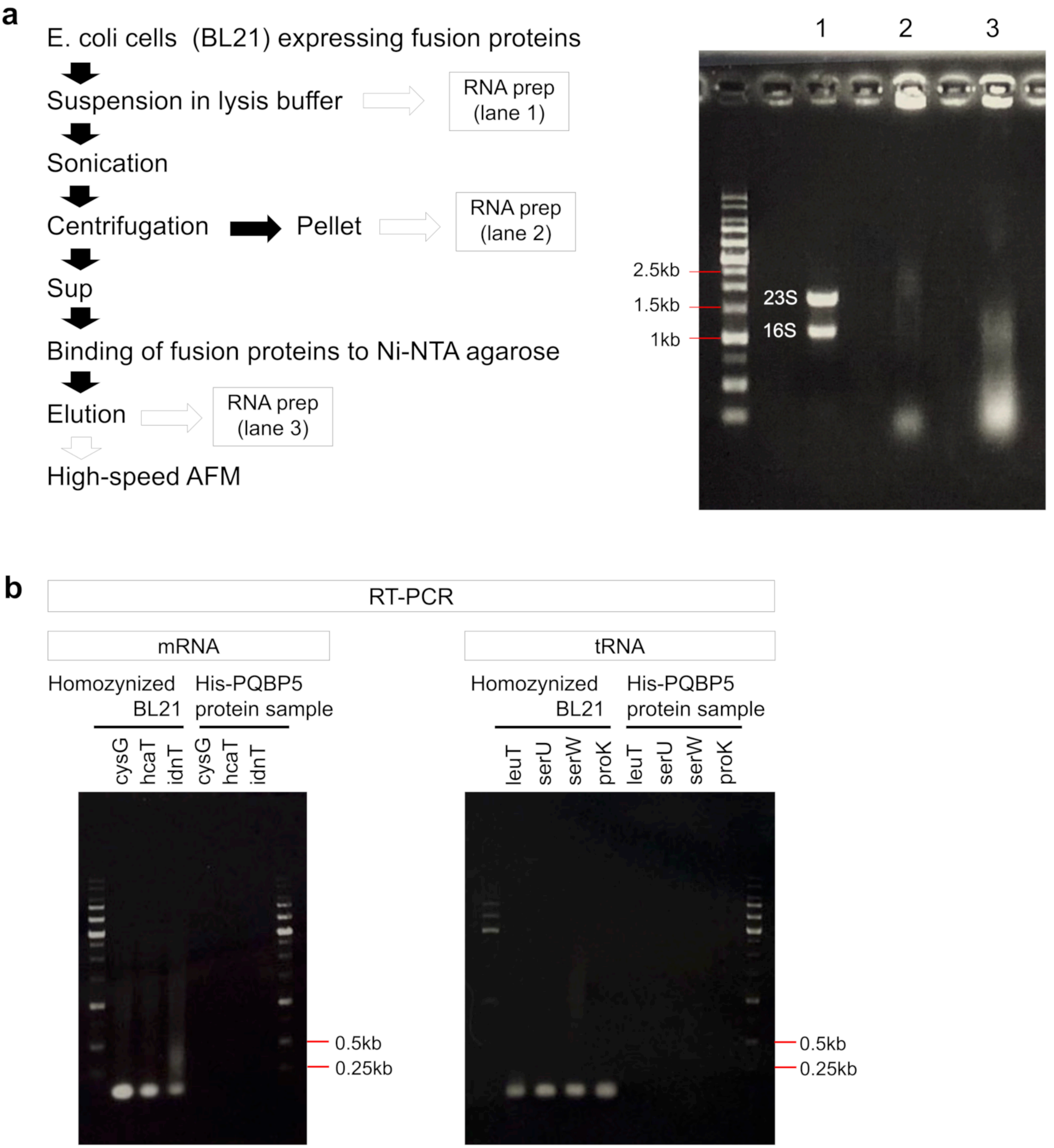

Supplementary Figure 16
Analysis of RNA interacting with PQBP5 in AFM
a) Left panels show the method of purification of His-tag-PQBP5 and origins of RNAs extracted by RNeasy kits. RNA extracted from purified His-PQBP5 protein (lane 3) was electrophoresed with RNAs from intermediates during purification process (lane 1, 2). The similar experiment was repeated three times.
b) RT-PCR showing contamination of mRNA of control genes (cysG, hcaT, idn T) or tRNA of control genes (LeuT, SerU, SerW, ProK) in protein samples for AFM (His-PQBP5 protein sample). RT-PCR for the same mRNAs and tRNAs were performed as positive controls. The similar experiment was repeated three times.
