# Supplementary material for: PQBP5/NOL10 maintains and anchors the nucleolus under physiological and osmotic stress conditions
Source: Nat Commun. 2023 Jan 4;14:9. doi: 10.1038/s41467-022-35602-w (PMC9813255; doi:10.1038/s41467-022-35602-w)
Supplement: Supplementary file 3 — Description of Additional Supplementary Files [file 41467_2022_35602_MOESM3_ESM.pdf]

Description of Additional Supplementary Files

File name: Supplementary Movie 1
Description: High-speed AFM of PQBP5
PQBP5 shows dynamic changes in the C-terminal region from a linear to a globular structure.

File name: Supplementary Movie 2
Description: High-speed AFM of PQBP5 interacting with RNA #1
PQBP5 contacts with RNA via a globular structure transiently formed in the middle part.

File name: Supplementary Movie 3
Description: High-speed AFM of PQBP5 interacting with RNA #2
PQBP5 contacts with RNA via a globular structure transiently formed in the distal part.

File name: Supplementary Movie 4
Description: 3D reconstruction of a nucleolus generated by Imaris from 2D images of super resolution microscopy
3D structure of a nucleolus composed of PQBP5 (green), fibrillarin (red) and nucleolin (light blue). PQBP5 formed a type of meshwork that could constitute the skeleton or frame of the nucleolus. By contrast, nucleolin formed peripheral condensates, and fibrillarin formed granular structures inside the nucleolus.

File name: Supplementary Movie 5
Description: 3D image of nucleoli generated by Zen from data obtained by LSM980 with Airyscan 2 (Carl Zeiss Co., Ltd).
3D structure of nucleoli composed of PQBP5 (green), fibrillarin (red) and nucleolin (light blue).

File name: Supplementary Movie 6

Description: 3D image of a nucleolus generated by Zen from data obtained by Elyra 7 (Carl Zeiss Co., Ltd).

3D structure of a nucleolus composed of PQBP5 (green), fibrillarin (red) and nucleolin (light blue).

File name: Supplementary Movie 7

Description: 3D image of a nucleolus generated by MetaMorph from data obtained by SRM equipped with CSU-W1 SoRa (Yokogawa Electronic Corporation)

3D structure of a nucleolus composed of PQBP5 (green), fibrillarin (red) and nucleolin (light blue).
