# Supplementary material for: PQBP5/NOL10 maintains and anchors the nucleolus under physiological and osmotic stress conditions
Source: Nat Commun. 2023 Jan 4;14:9. doi: 10.1038/s41467-022-35602-w (PMC9813255; doi:10.1038/s41467-022-35602-w)
Supplement: Supplementary file 11 — Reporting Summary [file 41467_2022_35602_MOESM11_ESM.pdf]

nature portfolio

Corresponding author(s): Hitoshi Okazawa

Last updated by author(s): Dec 3, 2022

Reporting Summary

Nature Portfolio wishes to improve the reproducibility of the work that we publish. This form provides structure for consistency and transparency in reporting. For further information on Nature Portfolio policies, see our Editorial Policies and the Editorial Policy Checklist.

Statistics

For all statistical analyses, confirm that the following items are present in the figure legend, table legend, main text, or Methods section.

n/a	Confirmed	
☐	☒	The exact sample size (*n*) for each experimental group/condition, given as a discrete number and unit of measurement
☐	☒	A statement on whether measurements were taken from distinct samples or whether the same sample was measured repeatedly
☐	☒	The statistical test(s) used AND whether they are one- or two-sided<br>*Only common tests should be described solely by name; describe more complex techniques in the Methods section.*
☐	☒	A description of all covariates tested
☐	☒	A description of any assumptions or corrections, such as tests of normality and adjustment for multiple comparisons
☐	☒	A full description of the statistical parameters including central tendency (e.g. means) or other basic estimates (e.g. regression coefficient) AND variation (e.g. standard deviation) or associated estimates of uncertainty (e.g. confidence intervals)
☐	☒	For null hypothesis testing, the test statistic (e.g. F, t, r) with confidence intervals, effect sizes, degrees of freedom and P value noted<br>*Give P values as exact values whenever suitable.*
☒	☐	For Bayesian analysis, information on the choice of priors and Markov chain Monte Carlo settings
☒	☐	For hierarchical and complex designs, identification of the appropriate level for tests and full reporting of outcomes
☒	☐	Estimates of effect sizes (e.g. Cohen's *d*, Pearson's *r*), indicating how they were calculated

Our web collection on statistics for biologists contains articles on many of the points above.

Software and code

Policy information about availability of computer code

Data collection	3D images were obtained with Imaris (OXFORD instruments), Zen (Carl ZEISS) or MetaMorph (MOLECULAR DEVICES).<br>ImageJ ver.1.53 (NIH) was used for 2D image processing and acquisition of signal intensity.<br>Protein Thermal Shift Software v1.4 (#4466037, Thirmo Fisher Scientific) was used to perform thermal shift assay.
Data analysis	GraphPad Prism 8 software (GraphPad Software, La Jolla, CA, USA) was used for statistical analyses and to plot data.<br>Microsoft Excel for Microsoft 365 was used to draw graphs.<br><br>A custom code for confirmation of SNP/GWAS data and polyQ disease association are available at https://doi.org/10.5281/zenodo.7389755.

For manuscripts utilizing custom algorithms or software that are central to the research but not yet described in published literature, software must be made available to editors and reviewers. We strongly encourage code deposition in a community repository (e.g. GitHub). See the Nature Portfolio guidelines for submitting code & software for further information.

Data

Policy information about availability of data

All manuscripts must include a data availability statement. This statement should provide the following information, where applicable:

- Accession codes, unique identifiers, or web links for publicly available datasets
- A description of any restrictions on data availability
- For clinical datasets or third party data, please ensure that the statement adheres to our policy

We wrote the following sentences in "Data Availability" section.

SNPs of PQBP5 were collected NCBI SNP database (https://www.ncbi.nlm.nih.gov/snp/).
Search results of SNP/GWAS data and polyQ disease-associated research papers are available at https://doi.org/10.5281/zenodo.7389755.
All other data generated or analyzed during this study are included in this article. Source data are provided with this paper.

Human research participants

Policy information about studies involving human research participants and Sex and Gender in Research.

Reporting on sex and gender	Not applicable.
Population characteristics	Not applicable.
Recruitment	Not applicable.
Ethics oversight	Not applicable.

Note that full information on the approval of the study protocol must also be provided in the manuscript.

Field-specific reporting

Please select the one below that is the best fit for your research. If you are not sure, read the appropriate sections before making your selection.

☒ Life sciences ☐ Behavioural & social sciences ☐ Ecological, evolutionary & environmental sciences

For a reference copy of the document with all sections, see nature.com/documents/nr-reporting-summary-flat.pdf

Life sciences study design

All studies must disclose on these points even when the disclosure is negative.

Sample size	No sample size calculation was performed, and the sample size were similar to those reported in previous publications, PubMed ID 29397273, 27641503 and 34980925.
Data exclusions	There are no exclusion criteria for all analysis.
Replication	Experiments were independently repeated, the numbers of biological replicates are presented in the Figures.
Randomization	Simple randomization was performed to allocate samples and/or images to researchers before analysis.<br>The selection of images from immunohistochemistry/immunocytochemisty and the actual experiments of IHC/ICC were done by different researchers. In vitro live-cell imaging were done by different researchers.<br>Western blots are repeated until the necessary N was acquired.
Blinding	The information about group allocation or samples were opened to the data analyst or image acquisition researchers after finalizing results (make graphs etc).

Reporting for specific materials, systems and methods

We require information from authors about some types of materials, experimental systems and methods used in many studies. Here, indicate whether each material, system or method listed is relevant to your study. If you are not sure if a list item applies to your research, read the appropriate section before selecting a response.

Materials & experimental systems

n/a	Involved in the study
☐	☒ Antibodies
☐	☒ Eukaryotic cell lines
☒	☐ Palaeontology and archaeology
☐	☒ Animals and other organisms
☒	☐ Clinical data
☒	☐ Dual use research of concern

Methods

n/a	Involved in the study
☒	☐ ChIP-seq
☒	☐ Flow cytometry
☒	☐ MRI-based neuroimaging

Antibodies

Antibodies used

All antibodies used in the study are listed in the method.
The antibodies used for immunoprecipitation were dilution as following, rabbit anti-FLAG antibody (#F7425, SIGMA, St. Louis, MO, USA); mouse anti-Myc antibody (#M047-3, MBL, Aichi, Japan); rabbit anti-fibrillarin antibody (1:1000 #ab166630, Abcam, Cambridge, UK); rabbit anti-nol10 antibody (1:5000 #ab181161 Abcam, Cambridge, UK); Mouse anti-nucleolin antibody (1:5000 #ab13541 Abcam, Cambridge, UK).
The antibodies used for Super resolution microscopy, Correlative light and electron microscopy, Osmotic stress and Immunocytochemistry were dilution as following, mouse anti-fibrillarin (1:100, #ab4566, Abcam, Cambridge, UK); rabbit anti-nol10/PQBP5 (1:150 #ab181161 Abcam, Cambridge, UK); mouse anti-nucleolin antibody (1:2000 #ab13541 Abcam, Cambridge, UK); mouse anti-NPM1 (1:5000, #ab10530, Abcam); rat anti-PES1 antibody (1:2000, #252849, Abcam); Alexa Fluor 488-conjugated anti-rabbit IgG (1:1000, #A21206, Molecular Probes, Eugene, OR, USA); Alexa Fluor 555-conjugated anti-mouse IgG (1:1000, #A31570, Molecular Probes, Eugene, OR, USA); donkey anti-mouse IgG (H+L) Highly Cross-Adsorbed Alexa Fluor 647 (1:1000, #31571, Thermo Fisher Scientific, Waltham, MA, USA); donkey anti-rat IgG (H+L) Highly Cross-Adsorbed Alexa Fluor 488 (1:1000, #21208, Thermo Fisher Scientific, Waltham, MA, USA); donkey anti-rabbit IgG (H+L) Highly Cross-Adsorbed Alexa Fluor 568 (1:1000, #10042, Thermo Fisher Scientific, Waltham, MA, USA).
The antibodies used for Western Blot were dilution as following, rabbit anti-fibrillarin (1:1000, #ab166630, Abcam, Cambridge, UK); mouse anti-C23 (1:5000, #sc-8031, Santa Cruz Biotechnology, Dallas, TX, USA); rabbit anti-nol10 (1:5000 #ab181161, Abcam, Cambridge, UK); rabbit anti-GFP (1:6000, #8334, Santa Cruz Biotechnology, Dallas, TX, USA); mouse anti-GAPDH (1:6000, #MAB374, Millipore, MA, USA). HRP-linked anti-rabbit IgG (1:3000, #NA934, GE Healthcare, IL, USA); HRP-linked anti-mouse IgG (1:3000, #NA931, GE Healthcare Life Sciences, Chicago, IL, USA).
The antibodies used for Immuno-electron microscopy were dilution as following, rabbit anti-GFP antibody (1:10000, #sc-8334, Santa Cruz Biotechnology, Dallas, TX, USA), Nanogold®-IgG goat anti rabbit IgG (H+L) (1:200, #2003, Nanoprobes, Yaphank, NY, USA); rabbit anti-FLAG antibody (1:250, #F7425, SIGMA, St. Louis, MO, USA); mouse anti-Myc antibody (1:250, #M047-3, MBL, Aichi, Japan).

The antibodies used for Immunohistochemistry were dilution as following, rabbit anti-NOL10 antibody (1:5000, #ab181161, Abcam); mouse anti-fibrillarin antibody (1:100, #ab4566, Abcam); mouse anti-nucleolin antibody (1:5000, #ab13541, Abcam); mouse anti-Htt antibody (1:100, MAB5374, Sigma Aldrich); mouse anti-Atxn1 antibody (1:50, NABN37, Sigma Aldrich); Alexa Fluor 488-conjugated anti-rabbit IgG (1:1000, A21206, Molecular Probes); Alexa Fluor 555-conjugated anti-mouse IgG (1:1000, #A31570, Molecular Probes).

Validation

Activities of rabbit anti-FLAG antibody is validated for all species via https://www.sigmaaldrich.com/JP/ja/product/sigma/f7425.
Activities of mouse anti-Myc antibody is validated for 6myc-tag fusion protein via https://www.mblintl.com/products/m047-3/.
Activities of rabbit anti-fibrillarin antibody is validated for human via https://www.abcam.co.jp/recombinant-human-dpy19l2-protein-ab166130.html.
Activities of rabbit anti-NOL10 antibody is validated for human via https://www.abcam.co.jp/nol10-antibody-epr14073-9-ab181161.html.
Activities of mouse anti-Nucleolin antibody is validated for human and mouse via https://www.abcam.co.jp/nucleolin-antibody-4e2-chip-grade-ab13541.html.
Activities of mouse anti-fibrillarin antibody is validated for mouse, rat and human via https://www.abcam.co.jp/fibrillarin-antibody-38f3-nucleolar-marker-ab4566.html.
Activities of mouse anti-NPM1 antibody is validated for mouse, rat, cow, dog, human, african green monkey and chinese hamster via https://www.abcam.co.jp/nucleophosmin-antibody-fc82291-ab10530.html.
Activities of rat anti-PES1 antibody is validated for mouse and human via https://www.abcam.co.jp/pescadillo-antibody-8e9-ab252849.html.
Activities of mouse anti-C23 antibody is validated for mouse, rat and human via https://www.scbt.com/ja/p/c23-antibody-ms-3.
Activities of rabbit anti-GFP antibody is validated for Green fluorescent protein via https://www.scbt.com/p/gfp-antibody-fl?requestFrom=search.
Activities of mouse anti-GAPDH antibody is validated for canine, human, mouse, rat, rabbit, fish, feline and pig via https://www.merckmillipore.com/JP/ja/product/Anti-Glyceraldehyde-3-Phosphate-Dehydrogenase-Antibody-clone-6C5,MM_NF-MAB374.
Activities of mouse anti-huntingtin antibody is validated for human, rat and mouse via https://www.merckmillipore.com/JP/ja/product/Anti-Huntingtin-Protein-Antibody-clone-mEM48,MM_NF-MAB5374.
Activities of mouse anti-ataxin1 antibody is validated for rat and mouse via https://www.merckmillipore.com/JP/ja/product/Anti-Ataxin-1-Antibody-11NQ-clone-N76-8,MM_NF-MABN37.

Eukaryotic cell lines

Policy information about cell lines and Sex and Gender in Research

Cell line source(s)

U2OS cells were kindly gifted from Dr. Yoshio Miki (Tokyo Medical and Dental University) that were purchased from ATCC (Manassas, VA, USA).

	HeLa cells were kindly gifted from Dr. Naoyuki Kataoka (Tokyo University) that were purchased from RIKEN BRC Cell Bank (Tsukuba, Japan).<br>HEK293 cells were purchased from TaKaRa (Kusatsu, Shiga, Japan).
Authentication	None of cell lines used were authenticated.
Mycoplasma contamination	All cell lines were negative for mycoplasma contamination.
Commonly misidentified lines<br>(See ICLAC register)	We did not use any misidentified cell lines.

Animals and other research organisms

Policy information about studies involving animals; ARRIVE guidelines recommended for reporting animal research, and Sex and Gender in Research

Laboratory animals	Mutant Ataxin-1 knock-in mice (Sca1154Q/2Q mice) were a generous gift from Prof. Huda Y. Zoghbi (Baylor College of Medicine, Houston, TX, USA)101, and mutant Huntingtin knock-in mice (HdhQ111 mice) were a generous gift from Prof. Marcy MacDonald (Massachusetts General Hospital, Harvard Medical School, Boston, MA, USA)102. C57BL/6J (BL/6) mice were used for breeding. Non-transgenic sibling mice were used as controls.<br>Mice were kept in 12 light/12 dark cycle, maintained at 20-22 degree with 40-60% humidity. Mice can access to food and water ad libitum.
Wild animals	The study did not involve any wild animals.
Reporting on sex	Both male and female mice were studied and compared in each of the experiments reported in this manuscript.
Field-collected samples	The study did not involve any samples collected from the field.
Ethics oversight	All animal experiments were performed in accordance with Animal Research: Reporting in vivo Experiments (ARRIVE) guidelines and were approved by the Committees on Gene Recombination Experiments and Animal Experiments of Tokyo Medical and Dental University (G2018-082C3 and A2021-211A).

Note that full information on the approval of the study protocol must also be provided in the manuscript.
